# Selective attention modulates neural envelope tracking of informationally masked speech in healthy older adults

**DOI:** 10.1002/hbm.25415

**Published:** 2021-03-30

**Authors:** Ira Kurthen, Jolanda Galbier, Laura Jagoda, Pia Neuschwander, Nathalie Giroud, Martin Meyer

**Affiliations:** ^1^ Developmental Psychology: Infancy and Childhood, Department of Psychology University of Zurich Zurich Switzerland; ^2^ Neuropsychology, Department of Psychology University of Zurich Zurich Switzerland; ^3^ Department of Computational Linguistics, Phonetics and Speech Sciences University of Zurich Zurich Switzerland; ^4^ Cognitive Psychology Unit Institute of Psychology, University of Klagenfurt Klagenfurt Austria; ^5^ University Research Priority Program “Dynamics of Healthy Aging”, University of Zurich Zurich Switzerland

**Keywords:** electroencephalography, energetic masking, envelope tracking, informational masking, older adults, speech processing, speech‐in‐noise

## Abstract

Speech understanding in noisy situations is compromised in old age. This study investigated the energetic and informational masking components of multi‐talker babble noise and their influence on neural tracking of the speech envelope in a sample of healthy older adults. Twenty‐three older adults (age range 65–80 years) listened to an audiobook embedded in noise while their electroencephalogram (EEG) was recorded. Energetic masking was manipulated by varying the signal‐to‐noise ratio (SNR) between target speech and background talkers and informational masking was manipulated by varying the number of background talkers. Neural envelope tracking was measured by calculating temporal response functions (TRFs) between speech envelope and EEG. Number of background talkers, but not SNR modulated the amplitude of an earlier (around 50 ms time lag) and a later (around 300 ms time lag) peak in the TRFs. Selective attention, but not working memory or peripheral hearing additionally modulated the amplitude of the later TRF peak. Finally, amplitude of the later TRF peak was positively related to accuracy in the comprehension task. The results suggest that stronger envelope tracking is beneficial for speech‐in‐noise understanding and that selective attention is an important ability supporting speech‐in‐noise understanding in multi‐talker scenes.

## Graphical abstract

This study investigated the energetic and informational masking components of multi‐talker babble noise and their influence on neural tracking of the speech envelope in a sample of healthy older adults. The results suggest that stronger envelope tracking is beneficial for speech‐in‐noise understanding and that selective attention is an important ability supporting speech‐in‐noise understanding in multi‐talker scenes.

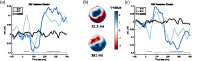

## 1 INTRODUCTION

Older adults often report difficulties in understanding speech in background noise (CHABA, 1988; Humes et al., 2012; Pichora‐Fuller, 1995), even when they are considered normal hearing based on pure‐tone thresholds (Füllgrabe, Moore, & Stone, 2015). Speech understanding difficulties are augmented in the presence of age‐related hearing loss (ARHL; Kortlang, Mauermann, & Ewert, 2016). ARHL, or presbycusis, is the most common form of sensorineural hearing loss, and it is one of the most prevalent age‐related conditions, estimated at approximately 20% at age 60, 50% at age 70, and 70–80% at age 80 and older (Bisgaard & Ruf, 2017; Goman & Lin, 2016). It can result in multiple unwanted outcomes like social isolation (Ciorba, Bianchini, Pelucchi, & Pastore, 2012; Mick, Kawachi, & Lin, 2014; Weinstein & Ventry, 1982) and potentially cognitive decline (Maharani, Pendleton, & Leroi, 2019) and eventually dementia (Lin et al., 2011). It is assumed that the higher risk for social isolation stems at least partly from the deficits in speech‐in‐noise perception, given that social situations typically contain background noise, which renders the listening situation challenging. Therefore, understanding the processes and abilities that lead to successful speech‐in‐noise understanding in older adults is key to helping develop additional treatments for ARHL, which let older adults maintain their social relationships.

Indeed, one of the most difficult communication situations is listening to a single speaker in the presence of other talkers, which is known as the “cocktail party problem” (Cherry, 1953). Both the target signal (the speech signal the listener aims to attend to) and the noise (the speech signals of other talkers which the listener is trying to ignore) are speech signals. Hence, the frequency bands in which these signals contain energy will tend to overlap, a phenomenon which is commonly referred to as “energetic masking” (EM; Brungart, 2001). However, speech‐on‐speech masking presents an additional challenge that cannot be explained only by an overlap in energy frequency bands. This additional type of masking has been labeled “informational masking” (IM; Brungart, 2001), and it is notoriously difficult to define, the common ground of all definitions being that its masking properties are nonenergetic, that is, not explained by overlap in energy frequency bands (Durlach et al., 2003; Rosen, Souza, Ekelund, & Majeed, 2013; Shinn‐Cunningham, 2008).

The amount of IM depends on the similarity of target and distractor, and, consequently, it can be reduced by increasing the dissimilarity between target and background talkers, for example by moving the distractor talker to a different location (Kidd, Mason, Rohtla, & Deliwala, 1998) or by introducing sex differences between target and distracting talkers (Brungart, 2001). IM has also been shown to take more effect when the background talkers speak the same language as the target speaker than when they speak a foreign language (Brouwer, Van Engen, Calandruccio, & Bradlow, 2012; Garcia Lecumberri & Cooke, 2006; Rhebergen, Versfeld, & Dreschler, 2005; Van Engen & Bradlow, 2007). Additionally, IM increases when the distractor language is known to the listeners, compared to an unknown language (Garcia Lecumberri & Cooke, 2006). These results have been explained on the basis of an increased cognitive load because of language‐decoding mechanisms that take place when a known language is presented as distractor (Cooke, Garcia Lecumberri, & Barker, 2008). This conclusion has been strengthened by the finding that IM is stronger when distractor speech consists of meaningful as opposed to semantically anomalous sentences (Brouwer et al., 2012).

According to Shinn‐Cunningham (2008), IM may be related to failures of object‐based attention, either because of failures in object formation, which occur when separate sources in a scene cannot be separated from one another, or because of failures in object selection, which occur when top‐down attention is directed to the distractor rather than the target. Object formation may be more difficult for hearing‐impaired individuals because of a spectrotemporally degraded representation of the speech input (Shinn‐Cunningham & Best, 2008). This degraded representation results in perceptually more similar target and distractor objects. This similarity, in turn, leads to more difficulties in object selection (Shinn‐Cunningham & Best, 2008), which can explain speech understanding difficulties of hearing‐impaired individuals in multi‐talker scenes. The Framework for Understanding Effortful Listening (Pichora‐Fuller et al., 2016) comprehensively describes how attention governs the allocation of cognitive capacity to cope with difficult listening situations.

Another cognitive ability that is relevant for speech‐in‐noise understanding is working memory. Because masked speech as well as peripheral hearing loss can result in a degraded representation of the input signal, the role of working memory in phonological and lexical retrieval and in pattern matching as posited by the Ease of Language Understanding model (Rönnberg et al., 2013) is significant in a multi‐talker scene. Indeed, especially for older individuals, working memory capacity is a very reliable predictor for speech‐in‐noise understanding (Besser, Koelewijn, Zekveld, Kramer, & Festen, 2013; Moore et al., 2014; Zekveld, Rudner, Johnsrude, & Rönnberg, 2013), although for some counterevidence see Schoof and Rosen (2014).

The “effortfulness hypothesis” (e.g., McCoy et al., 2005) underscores the importance of working memory capacity as a pool of resources that can be spent either on processing the sensory input or performing higher‐level computations on that input. This effortfulness hypothesis (e.g., McCoy et al., 2005) poses that under adverse listening conditions, resources are spent on the processing of the challenging stimuli, which may later not be available for performing mental computations on the input, like encoding semantic content into memory. To measure performance in both of these domains (perceptual processing and semantic encoding) in our study, our participants had to complete two tasks: an *intelligibility task* (IT), which simply tested how well participants could follow the target speaker, and a *comprehension task (CT)*, which tested participants' memory of the semantic content of the target speech signal. If there are no differences in intelligibility, there might be differences in comprehension, depending on how many resources were spent during the earlier task. For similar theoretical accounts see Wayne and Johnsrude (2015) and Nixon, Sarant, and Tomlin (2019).

### 1.1 Age‐related changes in acoustic cue processing and neural envelope tracking

With speech being an acoustic signal, the way the processing of acoustic cues changes with age is also of considerable importance. In general, aging is accompanied by a slowing of many processes (Salthouse, 1996, 2000), and this does not seem to be different in the acoustic domain. The acoustic signature of speech can generally be divided into two parts: rapidly changing acoustic cues, the temporal fine structure, and slowly changing acoustic cues, the temporal envelope (e.g., Drullman, 1995; Shannon, Zeng, Kamath, Wygonski, & Ekelid, 1995; Smith, Delgutte, & Oxenham, 2002). Especially slowly changing envelope cues are crucial for successful speech understanding (Liem, Hurschler, Jäncke, & Meyer, 2014; Shannon et al., 1995). A number of studies have shown that while aging is accompanied by a decrease in the ability to process temporal fine structure, the ability to process slowly changing cues, like the temporal envelope, is preserved (Gordon‐Salant & Fitzgibbons, 1993; Schneider & Pichora‐Fuller, 2001; Wingfield, Lindfield, & Goodglass, 2000; Wingfield, Wayland, & Stine, 1992; Lorenzi, Gilbert, Carn, Garnier, & Moore, 2006; Schneider, Pichora‐Fuller, & Daneman, 2010; Meyer, Keller, & Giroud, 2018; Giroud, Keller, Hirsiger, Dellwo, & Meyer, 2019). In conclusion, it appears that slow acoustic features of speech are an important resource for older adults to draw upon when understanding speech, especially in challenging listening situations.

On a neural level, it is presumed that the initial encoding of speech occurs by means of entrainment of ongoing cortical oscillations to the speech envelope (e.g., Giraud & Poeppel, 2012; Gross et al., 2013). This entrainment to the envelope is also called “envelope tracking,” and studies with transcranial alternating current stimulation have provided evidence that it serves a causal role for (i.e., functionally contributes to) speech intelligibility (Riecke, Formisano, Sorger, Başkent, & Gaudrain, 2018; Wilsch, Neuling, Obleser, & Herrmann, 2018; Zoefel, Archer‐Boyd, & Davis, 2018). Envelope tracking is a robust phenomenon, demonstrated by two studies that found envelope tracking even in severe acoustic interference from a competing talker (SNR between attended and ignored talker up to −8 dB; Ding & Simon, 2012, 2013). Nevertheless, it exhibits considerable inter‐individual variability (Lam, Hultén, Hagoort, & Schoffelen, 2018).

When comparing envelope tracking in younger and older adults, older adults on average typically show a stronger cortical response than younger adults (Decruy, Vanthornhout, & Francart, 2019; Presacco, Simon, & Anderson, 2016b). Similarly, a neural over‐representation of the envelope compared with the temporal fine structure has been demonstrated in individuals with ARHL (Anderson, White‐Schwoch, Choi, & Kraus, 2013), probably because ARHL mainly occurs in higher frequencies, which leaves the envelope relatively intact. It is currently unclear whether stronger envelope tracking is adaptive and reflects compensatory mechanisms or whether it constitutes a true “over”‐representation, which can hinder processing of the temporal fine structure (Decruy et al., 2019). At least in a study with young adults, stronger envelope tracking was highly positively correlated with subjectively rated speech‐in‐noise intelligibility (Ding & Simon, 2013).

Envelope tracking is especially relevant in a cocktail‐party environment. A study by O'Sullivan et al. (2015) showed that envelope tracking predicted target speech intelligibility in a cocktail‐party situation. A study by Vander Ghinst et al. (2016) investigated how envelope tracking and the noise level of multi‐talker babble noise were related. They found that as the noise level increased, envelope tracking decreased. Selective attention, which is an important ability for auditory object formation and selection (Shinn‐Cunningham, 2008), is also important for envelope tracking. In a study by Kerlin, Shahin, and Miller (2010), selective attention was shown to increase the gain of ongoing speech representations in a cocktail party scenario. Zion Golumbic et al. (2013) showed that speech representations in cocktail party scenarios become more and more sharpened to the target speech while a sentence unfolds and while the signal progresses through the processing hierarchy. Entrainment of cortical oscillations to the speech envelope may even serve to suppress the competing speech signal (Horton, D'Zmura, & Srinivasan, 2013), which is an ability that seems to be impeded with higher levels of peripheral hearing loss (Petersen, Wöstmann, Obleser, & Lunner, 2017). Taken together, these results suggest that envelope tracking is a necessary step in speech processing, that envelope tracking can be increased by exerting selective attention, and that stronger envelope tracking is positively related with speech intelligibility in multi‐talker situations.

Although working memory is a reliable predictor of speech‐in‐noise understanding in behavioral studies, a recent study found only weak evidence for an involvement of working memory in speech envelope tracking (Decruy et al., 2019). However, that particular study and many others have measured envelope tracking by performing envelope reconstruction from observed brain activity using backward modeling. This method provides a quantification of envelope reconstruction fidelity, but it does not take into account temporal aspects of envelope tracking. In our study, we measured envelope tracking by fitting TRFs, which are also called auditory‐evoked spread spectrum analyses (AESPAs; Lalor, Power, Reilly, & Foxe, 2009), and which constitute a form of forward modeling that allows to observe how the tracking of the envelope unfolds across time. One can therefore specifically analyze envelope tracking in time windows during which cognition is known to influence brain activity (O'Sullivan et al., 2015). Power, Foxe, Forde, Reilly, and Lalor (2012) have used this method to detect an attention‐modulated peak in the AESPA at a lag of around 200 ms.

### 1.2 Study design and hypotheses

Reconciling the two main findings that cognitive ability and hearing loss are related to speech‐in‐noise understanding and that aging and selective attention modulate envelope tracking, it is useful to ask whether envelope tracking is a mechanism by which cognition exerts its positive influence on speech‐in‐noise understanding. Because the EM and IM components of multi‐talker babble noise represent different challenges during speech‐in‐noise processing, we aimed to ascertain whether and how EM and IM would affect envelope tracking. Because previous literature has reported interaction effects of EM and IM (Brungart, 2001; Rosen et al., 2013), we also aimed to investigate whether EM and IM affect envelope tracking independently or with additive effects. Finally, because cognitive abilities like selective attention and working memory provide resources to cope with speech‐in‐noise processing, we aimed to investigate how individual differences in cognition relate to envelope tracking.

To this end, we presented our participants with speech in multi‐talker babble noise while their electroencephalogram (EEG) was recorded. To create acoustic conditions with different levels of EM and IM, we manipulated the SNR between target speaker and background noise for EM and the number of background talkers (nBTs) for IM, because previous research on IM arising from lexical interference has shown that IM decreases when nBT increases (Rosen et al., 2013).

To account for speech processing difficulties that would propagate beyond mere speaker tracking (effortfulness hypothesis; McCoy et al., 2005), participants had to complete two tasks: an intelligibility task and a CT. If there are no differences in performance in the intelligibility task between conditions, there might be differences in performance in the CT that emerge because of different amounts of resources remaining, after more or less of them were spent on processing the input, but not working on it or storing it.

To extract neural envelope tracking from the EEG recordings, we fitted temporal response functions (TRFs) to the envelope of the target signal with functions provided by the mTRF toolbox for MATLAB (Crosse, Di Liberto, Bednar, & Lalor, 2016). TRFs are forward models of the “time course of the neural response evoked by a unit power increase of the stimulus” (Ding & Simon, 2012, p. 11856), and they contain timing and spatial information of the neural encoding process (Ding & Simon, 2012). In fact, they are (and look) similar to auditory event‐related potentials (ERPs, Lalor et al., 2009), although they only represent neural activity in reaction to a specific feature, in our case the envelope, and not the net sum of all activity that is time‐locked to stimulus onset. As such, TRFs are ideally suited to quantify envelope tracking.

In a magnetencephalography study featuring an attended‐speech paradigm, Ding and Simon (2013) found that amplitude of an early neuromagnetic component “M50” of the TRF linearly decreased with increasing noise level, but the amplitude of a later component “M100” of the TRF was not affected by noise until it drastically decreased between −6 and −9 dB. Ding and Simon (2012) found that the M100 peak of the TRF was modulated by attention, whereas the M50 was not. Also, up to −8 dB SNR, there was no effect of SNR on peak amplitude. However, Petersen et al. (2017) found that differences in SNR resulted in differences in envelope tracking, with tracking of attended speech being stronger in lower noise levels than in higher noise levels. Petersen et al. (2017) presented speech in subject‐specific SNRs, while Ding and Simon (2012) and Ding and Simon (2013) used absolute SNRs. We also used absolute SNRs because we aimed to exclude any possible interference effects of EM and IM (Rosen et al., 2013) that could emerge differentially with subject‐specific SNRs.

While earlier ERPs are associated with perceptual processing of exogenous stimuli, later peaking ERPs indicate cognitive and endogenous processing, like for example the P3b (Giroud, Lemke, Reich, Matthes, & Meyer, 2017; van Dinteren, Arns, Jongsma, & Kessels, 2014). Because EM is mainly a perceptual interference, we hypothesized that differences in SNR would manifest at early time points. Because IM due to lexical interference should tap higher‐order cognitive resources, we hypothesized differences between TRFs in response to different numbers of background talkers during a later time window typically associated with cognitive processing.

Because selective attention has been shown to increase the gain of ongoing speech representations during multi‐talker babble noise (Kerlin et al., 2010), we hypothesized that a measurement of participants' ability to exert selective attention would predict envelope tracking. Exploratively, because working memory is the most commonly found predictor for speech‐in‐noise understanding, we also tested whether working memory would predict envelope tracking. Finally, because envelope tracking has been shown to be related to hearing thresholds (Petersen et al., 2017), we also tested whether hearing thresholds would predict envelope tracking.

## 2 MATERIALS AND METHODS

### 2.1 Participants

The sample consisted of 23 older adults (mean age = 70.96 years, *SD* = 3.72 years, 14 females). One more participant was tested but excluded due to technical issues during EEG recording. All participants were right‐handed as assessed by the Annett Hand Preference Questionnaire (Annett, 1970) and reported no psychiatric or neurological disorders. Their native language was Swiss German and they had not learned another language before their seventh year of age. They did not play music for more than 6 hours per week and they did not wear a hearing aid. Their hearing loss (pure‐tone average; PTA) did not exceed 60 dB in the frequencies 500, 1,000, 2,000, and 4,000 Hz, and the mean difference in hearing thresholds between the two ears did not exceed 20 dB. They passed a screening procedure, in which the exclusion criteria were tested via questionnaires and their hearing thresholds were measured with a MAICO ST‐20. PTA ranged from 10.44 to 50.63 dB HL. A Kolmogorov–Smirnov test against the normal distribution revealed that PTA was approximately normally distributed in our sample (*D* = 0.11371, *p* = .92). Additionally, participants were administered the Montreal Cognitive Assessment (Nasreddine et al., 2005) and were invited to further participate in the study when they scored 26 points or more.

The ethics committee of the Canton of Zurich approved the study (application no. 2017‐00284). Written informed consent was obtained from all participants. Participants were compensated for their participation.

### 2.2 Cognitive tests

Selective attention was measured with the Eriksen–Flanker Task (Eriksen & Eriksen, 1974), administered in a computer version modeled after the version reported in Stins, Polderman, Boomsma, and de Geus (2007) and available in the PEBL software, Version 0.14 (Mueller & Piper, 2014). From this task, we extracted each participant's Flanker score by subtracting their mean reaction time to the incongruent Flanker stimuli from their mean reaction time to the congruent Flanker stimuli. Lower scores indicate better selective attention.

Working memory was assessed with the Sentence Span task from the working memory capacity test battery by Lewandowsky, Oberauer, Yang, and Ecker (2010) implemented in MATLAB (The MathWorks, Inc., Natick, MA). Participants had to remember and recall letters in order of presentation. Letters were presented in set sizes of three to seven. Each set size occurred three times. As a distractor task, before each letter was presented, a sentence was displayed that had to be classified as “correct” or “false” (e.g., “The earth is larger than the sun.”). The difficulty in this distractor task was kept low because this level of difficulty had improved the correspondence between the Sentence Span measure and a latent measure of working memory capacity in a previous study (Lewandowsky et al., 2010 ). See Table 1 for a correlation matrix of age, PTA, and cognitive variables.

**TABLE 1:** Correlation matrix of age and envelope tracking predictors

	Age	PTA	Flanker
Age			
PTA	0.42*		
Flanker	−0.06	0.18	
Sentence span	0.03	−0.10	−0.11

*Note*: *p <.05, **p <.01, ***p <.001. Negative values indicate negative correlations. Note that for PTA, larger values indicate higher hearing thresholds; for Flanker, higher values indicate worse selective attention; for Sentence Span, higher values indicate better working memory.

### 2.3 Stimuli for EEG experiment

In the main EEG experiment, participants listened to natural speech in background noise that was made up of background talkers. The speech material was taken from a recording of a German audio book (*Die Glasglocke* by Sylvia Plath), recorded by a professional female speaker (F0 = 160.47 Hz, *SD* = 8.91 Hz). From this recording, we created the auditory stimuli for our EEG experiment. Stimuli were created to reflect different levels of EM and IM in a 2 × 2 design: To present different levels of EM, we manipulated the SNR between target speaker and background noise. To present different levels of IM, the nBTs was manipulated, as it has been shown that IM arising from lexical interference decreases when nBT increases (Rosen et al., 2013).

Because the addition of other talkers can also influence EM (Rosen et al., 2013), and because the effectiveness of IM strongly depends on similarity between talker and distractor (e.g., sex, semantic content, and spatial orientation), we decided to model the background noise from the same speaker who uttered the target signal. Furthermore, EM in multi‐talker babble noise strongly depends on whether the background talkers are currently speaking or pausing. To reduce opportunities for glimpsing, silent periods in the noise stimuli were trimmed. This way, we ensured that EM was always present and we can assume a monotonic increase in EM by lowering the SNR and a monotonic decrease in IM by adding more background talkers (Table 2).

**TABLE 2:** Summary statistics of hearing and cognitive abilities

	Original	*z*‐Scored
	(*N* = 23)	(*N* = 23)
*Hearing thresholds (PTA)*		
Mean (*SD*)	27.7 (9.34)	−0.02 (1.02)
Median [min, max]	26.5 [10.4, 50.6]	−0.30 [−1.71, 2.24]
*Working memory (sentence span)*		
Mean (*SD*)	0.60 (0.27)	−0.02 (1.02)
Median [min, max]	0.65 [0, 0.95]	−0.15 [−1.90, 2.48]
*Selective attention (flanker)*		
Mean (*SD*)	35.7 (48.5)	−0.03 (1.01)
Median [min, max]	22.0 [−44.8, 143]	0.15 [−2.28, 1.29]

*Note*: Summary statistics for hearing and cognitive ability scores. The first column shows the scores how they were obtained from the hearing and cognitive tasks, and the second column shows the z‐scored scores. Please note that the *z*‐scored scores do not have 0 as their mean and 1 as their standard deviation because *z*‐scoring was performed on data of all 24 initial participants. PTA, pure‐tone average.

For the nBT, we decided to present two conditions with two background talkers (2 BT), because this is the most difficult multi‐talker babble condition (Freyman, Balakrishnan, & Helfer, 2004; Rosen et al., 2013) and two conditions with eight background talkers (8 BT), because then, the background talkers mask one another and lexical interference is reduced. Different SNRs were tested in a pilot study, from which the SNRs 0 and 2 resulted. An overview of the experimental conditions can be found in Table 3.

**TABLE 3:** Experimental conditions

	Number of background talkers (IM)	
SNR (EM)	2	8
0	High EM, high IM	High EM, low IM
2	Low EM, high IM	Low EM, low IM

*Note*: This table shows the four experimental conditions. Each condition is defined by high or low EM and IM.

To create the stimuli, the full audio recording was manually split into segments that were coherent in content and had a length of about 45 s. Second, these longer segments were split into three shorter segments of about 15 seconds (mean duration = 14.69 s, *SD* duration = 3.46 s, min duration = 8.37 s, max duration = 28.54 s). Special care was taken to ensure that all of these three shorter segments ended with a full stop.

To create the speech‐in‐noise stimuli, we first trimmed all the silent periods with a duration longer or equal to 0.1 s in the longer sound segments. Afterward, we normalized the trimmed longer sound segments to 70 dB. Then, we mixed two or eight of these segments together to create background noise for the 2 and the 8 BT conditions. This mixture was then again normalized to 70 dB. After this, we manipulated the sound files to fade in over the first 1.5 s. Afterward, we mixed the background noise with the target speech segments at an SNR of either 0 or 2 so that the background noise would fade in after 2 s of only the target speaker talking. This progressive addition of the background noise was implemented because target and noise speech signals were voiced by the same female speaker, and participants would otherwise not have been able to follow the target signal. Finally, this mixture was again normalized to 70 dB.

To create probe stimuli for a pattern‐matching task, a short snippet of 0.3 s was extracted from the last sixth of each sound file. This way, we ensured that the participants would continually need to track the target speaker to correctly categorize the probe snippet. We also ensured that the probe snippet would contain continuous speech and not a pause in the speech signal.

### 2.4 Speech‐in‐Noise tasks

Participants completed four experimental blocks. Each block contained 39 trials of a single condition. Trial length varied between 10.37 and 30.54 s, dependent on the length of the sound segment presented. The mean trial duration was 16.69 s, with a standard deviation of 3.46 s. Special care was taken to ensure that trial length did not vary between conditions (*p*‐values of post‐hoc Tukey HSD tests comparing trial length of all conditions were all >0.33). A trial began with 2 s of silence. Next, the trial's sound segment was presented without background noise for 2 s, after which the background noise faded in and ramped up until it reached its maximum sound level 3.5 s after segment onset. The trial ended when the sound segment ended. An IT modeled after the pattern‐matching task of Liem et al. (2014) was implemented after the end of each trial. For the IT, a probe stimulus (the short sound snipped of 0.3 s duration) was played 1 s after the sound segment had finished. The probe stimulus was taken either from the sound segment or from one of the background talkers. By means of a mouse click, participants stated whether the probe had been taken from the to‐be‐attended sound segment or not. If participants did not answer for 3 s, the next trial began. Stimulus presentation was controlled via sound card (RME Babyface Pro, RME, Haimhausen, Germany) and stimuli were presented via a loudspeaker with linear frequency response (8030B Studio Monitor, Genelec, Iisalmi, Finland).

Each conglomerate of three IT trials was taken from one of the longer, coherent sound segments from the audio book. After these three IT trials, a CT trial followed. In the CT, participants answered a four‐alternative multiple choice question about the semantic content of the three previous IT trials. Only one of the four answer options was correct. Participants answered the question by means of a keyboard button press. The CT was untimed, so that participants would not feel rushed to answer the question.

### 2.5 EEG recording and preprocessing

Participants sat in an EEG cabin in front of a computer screen. After a short instruction in which they were shown their EEG on a screen and could try out blinking, closing their eyes and grinding their teeth, they were asked to refrain from moving as much as possible. Then, a total of 4 min of resting‐state EEG was recorded (2 min with eyes open, 2 min with eyes closed). In the eyes open condition, participants were asked to fixate a fixation cross on the computer screen.

The practice block contained three easy IT trials with 8 BT and an SNR of 5, followed by one CT question pertaining to the content of the three IT trials. In case the participant gave a wrong answer in the practice IT, that trial was repeated until the correct answer was given. After the practice session, participants were encouraged to attenuate or amplify the stimuli in order to ensure proper audibility. The original loudness of the stimuli was 70 dB SPL, and the range of attenuation/amplification across participants was −5 to 2 dB. Therefore, the final loudness ranged between 65 and 72 dB SPL. Critically, participants' attenuation/amplification was correlated with their PTA (*r* = −.56, *p* = .004), with participants with higher PTA aiming for louder stimuli. After stimulus loudness adjustment, participants completed four experimental blocks, one for each condition, each of which took about 15 min. Block order was counterbalanced between participants.

Participants' EEG was recorded continuously from 128 Ag/AgCl electrodes (BioSemi ActiveTwo, Amsterdam, The Netherlands) with a ActiveTwo AD‐box amplifier system (BioSemi ActiveTwo, Amsterdam, The Netherlands) and was digitized at a sampling rate of 512 Hz. The data were online band‐pass filtered between 0.1 and 100 Hz and impedances were reduced below 25 kΩ. Data were analyzed in MATLAB Release 2016b using the FieldTrip Toolbox (Oostenveld, Fries, Maris, & Schoffelen, 2011). For preprocessing, data were re‐referenced to Cz and then band‐pass filtered between 0.1 and 100 Hz with a noncausal zero‐phase two‐pass fourth order Butterworth IIR filter with −12 dB half‐amplitude cutoff. A noncausal zero‐phase two‐pass fourth order Butterworth IIR band‐stop filter with −12 dB half‐amplitude cutoff was applied between 48 and 52 Hz in order to eliminate artifacts resulting from electric interference. Data were visually screened for noisy channels, which were then removed. After that, the continuous EEG was segmented into trials starting 2 s before sound segment onset and lasting until the end of the sound segment (mean trial duration = 16.69 s, *SD* = 3.46 s, min trial duration = 10.37 s, max trial duration = 30.54 s). Trials containing gross artifacts were removed. After that, data were re‐referenced to an average reference and an independent component analysis (ICA; Jung et al., 2000) was applied. For the ICA, data were high‐pass filtered at 1 Hz in order to improve stationarity of the components. ICA components were inspected manually by two trained judges. ICA components were identified as representing blinks, saccades, muscle activity, or highly localized, singular artifacts based on topography, temporal occurrence, and frequency spectrum. After the removal of artefactual components, the remaining components were back‐projected to the original, 0.1‐Hz‐filtered data. On average, 8.55 components were removed per condition. Finally, noisy channels were interpolated using spline interpolation (Perrin, Pernier, Bertnard, Giard, & Echallier, 1987).

### 2.6 Temporal response functions

We fitted TRFs to the envelope of the target signal to extract neural envelope tracking from the EEG recordings. While cross‐correlating envelope and EEG (Petersen et al., 2017; Zoefel & VanRullen, 2016) is a similar approach to estimating the neural response to the envelope, TRFs are better suited because the speech envelope exhibits significant autocorrelation, which causes temporal smearing in the cross‐correlation approach (Crosse et al., 2016). We used the mTRF toolbox (Crosse et al., 2016) and followed the recommendations in the documentation in order to fit Temporal Response Functions (TFRs). In the mTRF toolbox, fitting of TRFs is achieved via ridge regression, which is among the best regularization methods for TRFs (Wong et al., 2018). We extracted the envelopes of the target speech signals with the *mTFRenvelope* function, downsampled them to 128 Hz and z‐scored them. After filtering the EEG between 1 and 15 Hz (as recommended by Crosse et al., 2016), we also downsampled the EEG of each trial to 128 Hz and z‐scored it. Additionally, the first 3.5 s of each EEG and envelope were removed because the background noise was either missing or ramping up during that time. Conveniently, this step also removed any activity in relation to early cortical evoked potentials. Out of a range of possible ridge values (*λ* = 2^0^, 2^2^, …, 2^20^), we identified the optimal ridge parameter to use for all participants, conditions, and channels via the *mTRFcrossval* function, using max *r* as optimization criterion. The optimal ridge parameter was *λ* = 2^8^. Then, a model for each participant, condition, and channel was trained via the *mTRFtrain* function, again with the *z*‐scored EEGs and envelopes. Each model was calculated over time lags between envelope and EEG between −150 and 450 ms, after which values for lags from −150 to −100 and from 400 to 450 ms were removed because of regression artifacts at the extremes of the models. For each trial, we additionally fitted TRFs between that trial's EEG and the envelope of the following trial (the EEG of each last trial per condition was paired with the envelope of the first trial). This was done in order to obtain baseline TRFs, which would be contrasted with the actual TRFs as a measurement for TRF quality.

### 2.7 Statistical analysis

Statistical tests were conducted in R, Version 3.6.2 (R Core Team, 2018) and FieldTrip, Version 20190419 (Oostenveld et al., 2011). The *p*‐values for estimates in linear mixed‐effects models (LMEM) were derived via the Satterthwaite method implemented in the R package *lmerTest* (Kuznetsova, Brockhoff, & Christensen, 2017).

All TRF analyses took place at lags between −100 and 400 ms. All statistical comparisons between conditions were conducted using cluster‐based permutation tests implemented in FieldTrip (Maris & Oostenveld, 2007). Dependent‐samples *t*‐tests were conducted for each sample between the TFRs of the two conditions to be compared. All samples whose *t*‐value exceeded a *p*‐value of .05 were clustered on the basis of temporal adjacency, with a cluster containing at least three neighboring channels. This was done separately for samples with negative and positive *t*‐values. The *t*‐values of each cluster were summed and the maximum absolute value of the cluster‐level statistics was taken. This maximum absolute value was then compared to a permutation distribution, which was obtained by randomly assigning the TFRs to the compared conditions, calculating the test statistic on this random set of trials and repeating this procedure 1,000 times. The critical *α* level for comparing the test statistic to the permutation distribution was set to .025 for a two‐sided *t*‐test (Maris & Oostenveld, 2007). All cluster‐based tests reported followed this procedure.

## 3 RESULTS

### 3.1 Behavioral results

**FIGURE 1:**
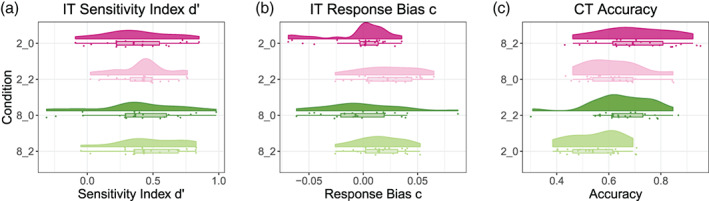
(a) Sensitivity index *d*′ by condition. (b) Response bias c by condition. (c) Accuracy in the CT by condition. CT, comprehension task; IT, intelligibility task; 2_0, 2 background talkers; SNR 0, 2_2, 2 background talkers; SNR 2, 8_0, 8 background talkers, SNR 0, 8_2, 2 background talkers, SNR 2

We first analyzed how EM and IM were related to performance in the two behavioral tasks. For the IT, which was a yes/no‐task, we estimated performance using Signal Detection Theory measures (Stanislaw & Todorov, 1999). The CT however was a four‐alternative forced choice task, and we therefore estimated performance using accuracy (the number of correct answers divided by the number of trials). See Figure 1 for a visualization of performance measures for the behavioral tasks. For the IT, we calculated the sensitivity index *d*′ and the response bias *c* (Stanislaw & Todorov, 1999) for each participant and for each of the four conditions. We first fitted a LMEM predicting *d*′ from SNR and nBT and their interaction, with a random intercept for participant (random slopes models did not converge). There was no significant effect of the condition factors nor their interaction. However, a LMEM predicting the response bias c from SNR and nBT and their interaction, with a random intercept for participant, revealed that SNR significantly predicted *c*, *b* = 0.03, *t*(66) = 4.39, *p* <.001, with SNR 2 resulting in a higher (i.e., more liberal) response criterion c than SNR 0. Both *d*′ and *c* were calculated using the *psycho* package for R (Makowski, 2018).

We also fitted a LMEM to the accuracy of the CT with SNR, number of talkers, and their interaction as predictors and a random intercept of participant. SNR significantly predicted accuracy in the CT, *b* = 0.10, *t*(66) = 3.19, *p* = .002, with higher accuracy for the SNR 2 conditions than for the SNR 0 conditions. Also, nBT significantly predicted accuracy in the CT, *b* = 0.06, *t*(69) = 2.02, *p* = .048, with a higher accuracy for the conditions with 8 BT than for the conditions with 2 BT. Therefore, both SNR and nBT influenced performance in the CT, but there was no evidence for an interaction effect of the two.

### 3.2 TRF results

**FIGURE 2:**
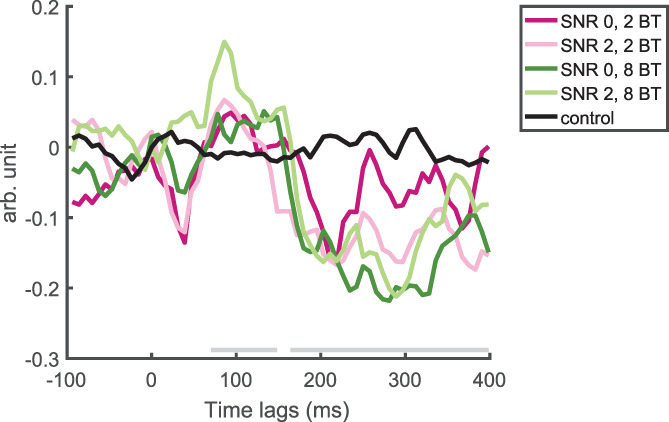
Grand average TFR traces of the four experimental conditions and the baseline TRF, averaged across postero‐occipital midline electrodes (A21, A22, A23, and A24), which are representative of the posterior electrode cluster. Time lags at which the average of the actual TRFs significantly differed from the baseline TRF (*p* <.05) are denoted with the gray bar slightly above the *x* axis. BT, background talkers; SNR, signal‐to‐noise ratio; TRF, temporal response function

Figure 2 shows the grand average TRFs for the four conditions and the grand average baseline TRF. Viewing the TRFs, in contrast to earlier MEG TRF estimations, we not only observed peaks in the TRF at around 50 and 100 ms, but also a third, prolonged peak, similar to the Pd in Power et al. (2012) and the *P*2_crosscorr_ in Petersen et al. (2017). In reference to the approximate timing of their maximum deflection, we will refer to them as TRF_50_, TRF_100_, and TRF_300_.

Using cluster‐based permutation tests, we first compared all TRFs to the baseline TRFs. All TRFs, when compared to the baseline separately, differed significantly in a first time window starting from ∼ 70 to ∼ 150 ms and in a second, longer time window, starting from ∼ 170 ms and lasting until the end of our time window. These differences were significant at almost all electrodes. We therefore concluded that TRF estimation had been successful in capturing brain activity related to the speech envelope. There was a small window between lags of around 150 and 170 ms where no significant difference was found. We suspect that this is due to the reversing of the polarity during this time window, which is bound to cross the zero line, around which the control TRFs hovered. Because sound onsets, which would have elicited auditory ERPs, had been removed from the EEG with which the TRFs were calculated, our TRFs mostly represent brain activity related to the tracking of the speech envelope (linearly; Crosse et al., 2016).

#### 3.2.1 TRFs as a function of SNR and nBT

Next, we compared the TRFs of conditions with SNR 0 to the TRFs of conditions with SNR 2. There was no significant difference at any time lag between the two conditions.

**FIGURE 3:**
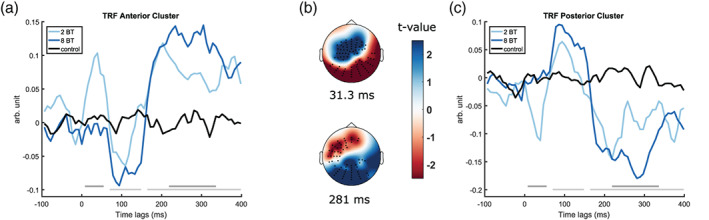
(a) Grand average TFR traces of the 2 BT conditions irrespective of SNR, the 8 BT conditions irrespective of SNR, and the baseline TRF, averaged across anterior cluster electrodes (C19, C25, C26, C27, C32, D3, D4, D5, D11, D12, D19, D20, and D27). Time lags at which the average of the actual TRFs differed from the baseline TRF are denoted with the light gray bar slightly above the *x* axis. Time lags at which the 2 BT TRFs differed from the 8 BT TRFs are denoted with the darker gray bar. (b) Topographies of *t*‐values at 31.3 and at 281 ms time lags. (c) Grand average TFR traces of the 2 BT conditions irrespective of SNR, the 8 BT conditions irrespective of SNR, and the baseline TRF, averaged across posterior cluster electrodes (A9, A10, A11, A12, A13, A14, A15, A16, A21, A22, A23, A24, A25, A26, A27, A28, A29, A31, A32, B3, B8, and B9). Time lags at which the average of the actual TRFs differed from the baseline TRF are denoted with the light gray bar slightly above the *x* axis. Time lags at which the 2 BT TRFs differed from the 8 BT TRFs are denoted with the darker gray bar. BT, background talkers; SNR, signal‐to‐noise ratio; TRF, temporal response function

Then, we compared the TRFs of the 2 BT conditions to the TRFs of the 8 BT conditions. There were two time lag windows at which the TRFs were significantly different; one early time window at lags of around 10–60 ms, reflecting TRF_50_ and one later time window at lags of around 210–330 ms, reflecting TRF_300_ (see Figure 3).

Inspection of the topography at time lags where significantly different effects were observed (also see Figure 3) revealed that these differences came about because of one positive and one negative cluster at each time lag. At each of the significantly different time windows, one negative and one positive cluster complemented each other, with one being located at left anterior and medial anterior electrodes and the other at postero‐occipital electrodes. Figure 3 shows the TRFs and topoplots of the clusters. Note that the terms “positive” and “negative” refer to the difference in *t*‐values between the conditions, and not to the polarity of the peaks associated with them. In fact, the positive clusters reflected negative peaks and the negative clusters reflected positive peaks in the TRFs. Because of that, more negative values in the positive clusters reflect larger amplitudes in the peaks associated with it, as do more positive values in the negative clusters.

As a next step, we tested the interaction effect of SNR and nBT. For that, we performed cluster‐based comparisons between the raw effect of SNR (difference in SNR TFRs) and the raw effect of nBT (difference in BT TRFs). There was no significant difference at any time lag in the interaction of the two conditions.

### 3.3 TRF cluster peak amplitudes as a function of SNR, nBT, hearing, and cognition

To test whether there was an association between TRF peak amplitudes and inter‐individual variables of hearing and cognition, we exported average values for the two positive and the two negative clusters that reached significance in the 2 versus 8 BT comparison. While reducing these multidimensional data to a single value means losing temporal and spatial information, it makes the fitting of more complex models feasible.

#### 3.3.1 Cooccurrence of clusters

First, we asserted whether the presumed association between the simultaneously occurring negative and positive clusters could be confirmed with the averaged values. To this end, we ran Pearson correlations between exported values of the positive and negative clusters of TRF_50_ and the positive and negative clusters of TRF_300_. The amplitudes in the TRF_50_ clusters were highly correlated (*r*[90] = −0.84, *p* <.001), as were the amplitudes in the TRF_300_ clusters (*r*[90] = −0.78, *p* >.001). These strong correlations support the view that positive and negative clusters occurring at the same time represent the same or at least related processes.

#### 3.3.2 TRF amplitude as a function of SNR and nBT

Second, we aimed to replicate the result of the FieldTrip analysis within R by fitting LMEMs with SNR and nBT and their interaction effect as fixed effects and a random intercept per participant (a random slopes model did not converge) to each of the four average cluster values. All four of the models contained a significant main effect of nBT (positive cluster of TRF_50_: *b* = −0.08, *t*(66) = −3.52, *p* <.001; negative cluster of TRF_50_: *b* = 0.08, *t*(66) = 3.01, *p* = .004; positive cluster of TRF_300_: *b* = −0.15, *t*(66) = −5.45, *p* <.001; negative cluster of TRF_300_: *b* = 0.12, *t*(66) = 5.27, *p* <.001), with the amplitude being higher in the 8 BT conditions than in the 2 BT conditions. Additionally, for the TRF_300_ positive cluster, there was a trend for a main effect of SNR, *b* = −0.05, *t*(66) = −2, *p* = .05, with a higher amplitude in the SNR 2 conditions than in the SNR 0 conditions.

Furthermore, for the TRF_300_ positive cluster, there was a significant interaction effect of SNR and nBT, *b* = 0.08, *t*(66) = 2.13, *p* <.04. Although amplitude was always higher in the 8 BT conditions than in the 2 BT conditions, the difference between the two was stronger when the SNR was 0 than when the SNR was 2.

#### 3.3.3 TRF amplitude as a function of SNR, nBT, and participant‐level variables

**FIGURE 4:**
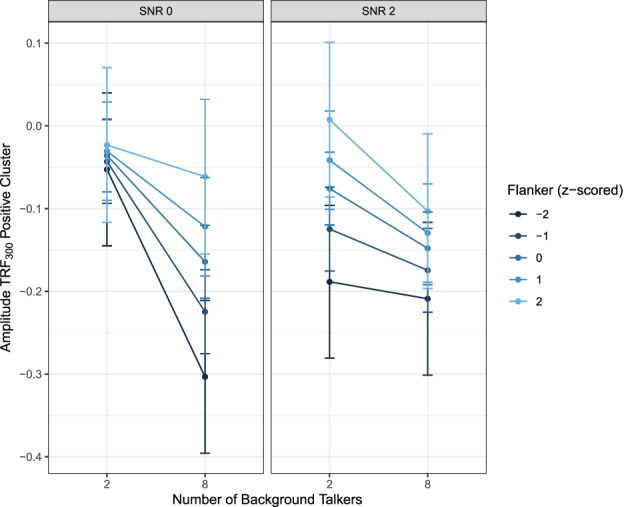
Effects plot of the three‐way interaction between SNR, nBT, and selective attention (as measured by the Flanker task). A lower Flanker score indicates better selective attention. Bars indicate 95% confidence intervals. nBT, number of background talkers; SNR, signal‐to‐noise ratio

In a third step, we updated the LMEMs with participant‐level variables. Specifically, we each added PTA, Sentence Span for working memory, and Flanker for selective attention as *z*‐scored predictors to the models separately. We then performed likelihood ratio tests between the models with and without participant‐level predictor. Neither the inclusion of PTA nor of working memory provided a significantly better fit to the data. The inclusion of selective attention provided a better fit to the data for models of the TRF_300_ positive cluster values, *χ* ^2^(4) = 11.90, *p* = .02, and negative cluster values, *χ* ^2^(4) = 10.24, *p* = .04. The inclusion of selective attention did not result in an additional significant effect in the model for the negative cluster values. For the positive cluster values, we found a three‐way interaction effect between SNR, nBT, and selective attention. Model parameters are reported in Table 4 and the effects are visualized in Figure 4. TRF_300_ amplitude was always larger in the 8 BT conditions than in the 2 BT conditions, but this difference was larger in the SNR 0 conditions than in the SNR 2 conditions. There was also a significant interaction effect between nBT and selective attention, with participants with better selective attention (lower *z*‐scores) having a stronger increase in amplitude between 2 and 8 BT. Regarding the three‐way interaction, in the SNR 0 conditions, better selective attention (lower *z*‐scores) led to a steeper decrease in TRF amplitude than worse selective attention. However, in the SNR 2 conditions, the increase in TRF_300_ amplitude between the 2 BT and the 8 BT conditions was steeper for participants with worse selective attention.

**TABLE 4:** Parameters from the model predicting TRF_300_ positive cluster amplitude from SNR, nBT, and selective attention

	Estimate	*SE*	*df*	*t*‐value	Pr (>|*t*|)	*SD* participant
(Intercept)	−0.038	0.021	73	−1.8	0.077	0.048
SNR2	−0.053	0.026	63	−2	0.05	
talkers8	−0.14	0.026	63	−5.5	7.4e−07	
Flanker_z	0.0074	0.021	73	0.35	0.73	
SNR2:talkers8	0.079	0.037	63	2.1	0.038	
SNR2:Flanker_z	0.042	0.026	63	1.6	0.11	
talkers8:Flanker_z	0.053	0.026	63	2	0.045	
SNR2:talkers8:Flanker_z	−0.076	0.037	63	−2.1	0.043	

*Note*: Model formula: amplitude – SNR * talkers * Flanker_z + (1 ∣ participant).

### 3.4 Task performance as a function of SNR, nBT, and cluster amplitudes

We further tested whether the inclusion of cluster amplitude improved the models for performance in the IT and the CT. To this end, we updated the previous models on the sensitivity index *d*′ in the IT and accuracy in the CT to include each of the positive and negative TRF_50_ and TRF_300_ clusters in separate models with full main and interaction effects with SNR and nBT. In total, 8 models were fitted. Again, we first tested whether this addition would provide a better fit to the data using likelihood ratio tests.

#### 3.4.1 IT task performance as a function of SNR, nBT, and cluster amplitudes

For the IT task, the inclusion of the TRF_300_ negative cluster into the model for the sensitivity index *d*′ provided a significantly better fit to the data than the basic model with just SNR and nBT, *χ* ^2^(4) = 12.63, *p* = .01. However, no additional significant effects were found in the model.

#### 3.4.2 CT task performance as a function of SNR, nBT, and cluster amplitudes

**FIGURE 5:**
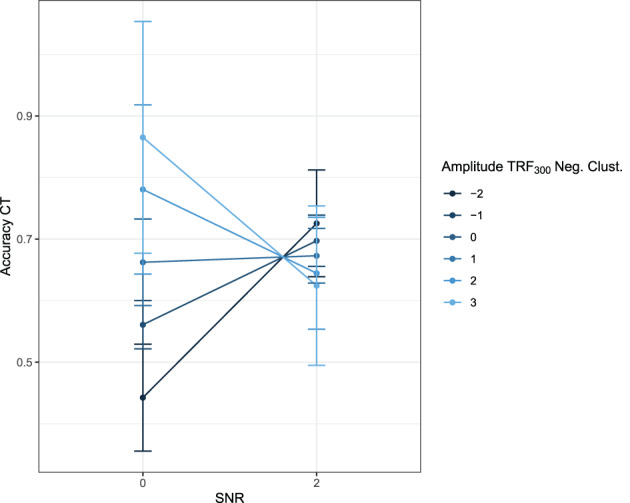
Effects plot of the interaction between SNR and amplitude of the TRF_300_ negative cluster. Bars indicate 95% confidence intervals. SNR, signal‐to‐noise ratio; TRF, temporal response function

For accuracy in the CT, the inclusion of the TRF_300_ positive cluster into the model provided a significantly better fit to the data than the basic model, *χ* ^2^(4) = 11.39, *p* = .02. However, this did not result in an additional significant effect in the model. The inclusion of the TRF_300_ negative cluster into the model for accuracy in the CT provided a significantly better fit, *χ* ^2^(4) = 15.24, *p* = .004. Amplitude of the TRF_300_ negative cluster significantly predicted accuracy in the CT, *b* = 0.13, *t* (83.17) = 2.59, *p* = .01, with a larger amplitude (more positive values) resulting in better accuracy. Additionally, there was a significant interaction effect between SNR and negative cluster amplitudes on CT task performance, *b* = −0.12, *t*(81.94) = −2.03, *p* = .046. This interaction effect is visualized in Figure 5. Amplitude of the TRF_300_ negative cluster predicted accuracy in the SNR 0 conditions, with a higher amplitude being related to higher accuracy, but not in the SNR 2 conditions.

## 4 DISCUSSION

This study aimed to investigate neural envelope tracking in multi‐talker babble noise in healthy older adults, how it is affected by varying degrees of EM and IM, and how peripheral hearing and cognition modulate envelope tracking in these situations. We found envelope tracking to be robust across conditions, indicating that envelope tracking took place regardless of the levels of EM and IM in the background noise. Nevertheless, in a time window typically associated with cognitive processing, stronger IM resulted in less envelope tracking. However, participants with better selective attention exhibited stronger envelope tracking, even in conditions with high IM. This finding underscores the importance of selective attention during speech‐in‐noise processing. The next sections will explore the results further and put them into the context of relevant literature.

### 4.1 Behavioral task performance

In our study, we measured speech understanding during speech‐in‐noise processing with two complementary tasks: the IT, which measured the ability to follow the target speaker, and the CT, which tested participants' memory of the lexical content of the target speech signal. These two tasks were conceived because of claims made by the effortfulness hypothesis (e.g., McCoy et al., 2005), which poses that resources which are spent on the processing of challenging stimuli (operationalized via the IT) may not be available later for performing mental computations on the input, like encoding semantic content into memory (operationalized via the CT).

Indeed, in our study, the ability to follow the target speaker was neither affected by EM nor by IM. We mainly observed effects of EM and IM in the CT, with higher SNR and higher number of talkers resulting in higher accuracy in the CT. In the IT, participants applied a more liberal response criterion (i.e., answered more with “yes”) to the SNR 2 conditions than to the SNR 0 conditions. Given our data, it is difficult to put into context why an SNR of 2 fostered that particular response pattern and whether the response bias is (non‐)linearly related to the SNR. Future studies should incorporate not only two different expressions of EM and IM each, but rather vary EM and IM as continuous variables in order to allow for the detection of possible nonlinear effects.

Nevertheless, our behavioral results can be viewed within the effortfulness hypothesis (e.g., McCoy et al., 2005), which posits decrements in performance not necessarily at early stages (here: following the target speaker in the IT), but when taxed with additional working memory load (answering content questions in the CT).

### 4.2 Envelope tracking in energetic and informational masking

The main research question of the study was how EM and IM independently and jointly influence envelope tracking in older adults. We hypothesized that differences in tracking due to increased EM, being mainly a perceptual interference, should manifest at early time points during tracking. On the other hand, differences in tracking due to increased IM, thereby tapping higher‐order cognitive resources, should manifest in a later time window typically associated with cognitive processing. With regard to EM, we found no effect of SNR on TRFs. Our results concur with those of a study that did not find an effect of SNR on TRFs as well (Ding & Simon, 2012). Although we had expected an early effect of SNR, as had been found in other studies, these studies either presented stimuli with very salient SNR differences (Ding & Simon, 2013) or presented SNR differences specific to participants' SRTs (Petersen et al., 2017). Possibly, with greater SNR differences, this effect might have emerged in our sample as well. Unfortunately, our SNR range was limited as pilot testing revealed that a higher SNR would have resulted in ceiling performance in the behavioral tasks. Indeed, when reducing the data to an average value across the time of the TRF_300_ peak, there was an almost significant trend (*p* = .05) for a main effect of SNR, although in a later time window than expected. Also, as more variables are introduced, SNR differences do seem to be meaningful, as will be explored in the corresponding sections of the discussion.

There were significant differences in the TRFs in the 2 versus 8 BT conditions. The TRF_50_ exhibited a larger amplitude in the 2 BT conditions than in the 8 BT conditions. For the sustained later peak, the TRF_300_, there was a larger amplitude in the 8 BT conditions than in the 2 BT conditions. In an attended/competing talker paradigm, Power et al. (2012) conducted a TRF/AESPA analysis and found an attention‐related component at around a lag of about 220 ms, the Pd, which was present for the speech envelope of an attended talker, but not for the envelope of the competing talker, and which they related to a semantic filtering process. Another study (Niemczak & Vander Werff, 2019) investigated how the P1‐N1‐P2 response was affected by EM and IM. In this study, IM was also manipulated by varying the nBT between 2 and 8 BT. They found a reduced P2 amplitude in the 2 BT condition compared to the 8 BT condition. Lalor et al. (2009) already assumed that the Pd (corresponding to our TRF_300_ cluster) might be related to the P2. Our results add to this body of research in supporting that a deflection in this time window might indeed reflect semantic filtering, based on the fact that our IM stimuli were constructed to reflect different amounts of perceivable semantic content.

The finding of the TRF_50_ cluster is especially interesting, because we did not expect such an early modulation of target signal envelope tracking between these two experimental conditions. By reducing glimpsing opportunities through elimination of silent periods in the background talkers' speech signals (see Section 2) and by creating background noise taken from the same speaker as the target speaker, we explicitly targeted lexical interference as the main component of interest of IM. Nevertheless, it is possible that stronger envelope tracking (as reflected in the larger TRF_50_) occurred in the 2 BT conditions because of remaining glimpsing opportunities. On another note, it might be useful to complement the analysis with an account of the auditory ERP component corresponding to the TRF_50_ with regard to temporal occurrence, the P50. The P50 is involved in sensory gating (Joos, Gilles, Van de Heyning, De Ridder, & Vanneste, 2014) and it has been suggested that top‐down modulation of sensory input is already present during such an early time window (Kurthen et al., 2007). Possibly, object formation (Shinn‐Cunningham, 2008) was more prominent in the 8 BT conditions because more auditory objects were present (there were nine instead of three speakers to encode), which in turn attenuated the response to each single speaker, including the target speaker.

The finding of the significant difference in the TRF_300_ cluster was expected because it occurred in a time window where an involvement of cognitive ability in stimulus processing had already been demonstrated (Giroud et al., 2017; Picton & Hillyard, 1974; Snyder, Alain, & Picton, 2006; van Dinteren et al., 2014). When visually examining the two TRF traces in Figure 3, it seems that the TRF_300_ is not only of reduced amplitude but also of shorter duration in the 2 BT conditions than in the 8 BT conditions. Given that in the study by Power et al. (2012), an AESPA peak at a similar latency signaled attention, this could reflect reduced attention directed at the target speaker in the 2 BT conditions. This interpretation is corroborated by the significant interaction between nBT and Flanker performance in the model that predicted the amplitude of the positive TRF_300_ cluster.

Using cluster‐based permutation tests, we found no interaction effect of SNR and nBT and thus no evidence that EM and IM add up in their effects on speech envelope tracking at any point.

### 4.3 TRF peak amplitudes as a function of experimental condition, hearing, and cognition

Because envelope tracking is assumed to take on a functional role for speech understanding (Riecke et al., 2018; Wilsch et al., 2018; Zoefel et al., 2018), it is important to investigate how envelope tracking is affected by inter‐individual participant characteristics. Identifying inter‐individual variables that influence envelope tracking might also explain differences in speech understanding.

In our study, we investigated how hearing thresholds, working memory, and selective attention influence speech envelope tracking. Neither hearing thresholds nor working memory were shown to predict envelope tracking. This was surprising given that hearing thresholds have been an important predictor for TRF peak amplitude in the study of Petersen et al. (2017). However, in their study, it predicted the amplitude of the *N*1_crosscorr_, which did not show up as a significantly different peak in our analysis and was therefore not subjected to such an analysis in our study. Additionally, we accounted for inter‐individual differences in hearing thresholds by allowing participants to alter the sound level of the stimuli. Because the hearing thresholds measured from our participants were sampled from a rather large range of hearing thresholds, between 10.44 (no clinical hearing loss) and 50.63 dB HL (moderate hearing loss), it is unlikely that the absence of an effect of PTA on TRF amplitude was due to a too short range of hearing thresholds in our participants. Possibly, hearing thresholds play a significant role in early, perceptual stages of speech processing, but not as much in later, cognitive stages. Consequently, counteracting peripheral hearing loss by means of a hearing aid might aid the early, perceptual processing of a speech signal, but might not be as effective in supporting the later, cognitive processing of speech in background talker noise (Bertoli et al., 2009).

Working memory is by far the most commonly found predictor for speech‐in‐noise processing (Besser et al., 2013; Moore et al., 2014; Zekveld et al., 2013). Because of its hypothesized role in cognitive processing of speech in noise (Rönnberg et al., 2013), we would have expected working memory to predict TRF amplitude at a later point in time. In the study by Decruy et al. (2019), working memory was positively related to envelope tracking in the presence of a competing talker. However, this positive relationship was found only in the context of a significant interaction effect between background noise type and working memory, and not as a main effect of working memory itself. Also, they used a backward modeling approach which allowed for a quantification of the reconstruction of the envelope, but not for its time course. Our approach of forward modeling allows for sample‐to‐sample comparison of conditions, and therefore enabled us to integrate previous knowledge of the involvement of cognition over the time course of speech processing into the investigation of EM and IM influences and thereby to identify two time windows (TRF_50_ and TRF_300_) during which differences in IM resulted in different amounts of envelope tracking. However, even with such temporal precision, there was no effect of working memory in our study. Our SNRs ranged from 0 to 2 dB, while in the study of Decruy et al. (2019), they ranged between 3 and −6 dB. Effects of working memory on envelope tracking between our conditions might have emerged with a larger difference in SNR.

The inclusion of selective attention as a variable provided a better fit to the data than just SNR and BT, but only for the TRF_300_ clusters. The three‐way interaction between SNR, nBT, and selective attention illustrates the nontriviality of combining EM and IM. The inclusion of selective attention also revealed an interaction between SNR and nBT, which indicated that while cluster amplitude was always larger in the 8 BT conditions than in the 2 BT conditions, this difference was larger in the SNR 0 conditions than in the SNR 2 conditions. Therefore, stronger EM led to a greater difference in envelope tracking in conditions varying in IM. There was also a significant interaction effect between nBT and selective attention, with participants with better selective attention having a stronger increase in TRF_300_ amplitude between the two conditions. The three‐way interaction revealed that this pattern was true only for the SNR 0 conditions, and that the increase in TRF_300_ amplitude between the two conditions was actually stronger for participants with worse selective attention in the SNR 2 conditions. These results can be interpreted as that the release from EM in the SNR 2 conditions played to the strengths of participants with lower selective attention ability, who, in the easier SNR conditions, could stronger differentiate between high and low IM. Contrary to our results, the study by Presacco, Simon, and Anderson (2016a) found that selective attention was negatively related to envelope tracking in older adults. However, they measured envelope tracking by means of envelope reconstruction fidelity. With our forward‐modeling approach, we could tap into envelope tracking during different time windows. Indeed, selective attention was only a relevant predictor in the later, TRF_300_ time window and not during the earlier TRF_50_ time window.

### 4.4 Behavioral relevance of envelope tracking

Finally, we were interested in whether envelope tracking served a functional role in speech understanding. We found envelope tracking in the later TRF_300_ time window to positively predict performance in the CT task, which measured how well participants could memorize the content of the target speaker's speech signal. Additionally, the significant interaction effect between envelope tracking and SNR revealed that envelope tracking was even more positively related to CT task performance in the more difficult SNR 0 condition. Therefore, stronger envelope tracking seems especially helpful in a more difficult listening situation in terms of EM.

Additionally, relative to young adults, older adults on average exhibit stronger envelope tracking and it has been debated whether this stronger envelope tracking is beneficial or hindering (Anderson, Parbery‐Clark, White‐Schwoch, Drehobl, & Kraus, 2013; Decruy et al., 2019). In our study, when looking at inter‐individual differences in older adults, stronger envelope tracking seems to be a factor that is beneficial for speech understanding in older adults. Specifically, it predicted accuracy in the CT, with which we assessed how well our participants memorized the content of the auditory stimuli. This result is in line with previous findings in young adults, where envelope tracking was positively related to speech‐in‐noise intelligibility (Ding & Simon, 2013). Possibly, a stronger representation of the envelope in neural activity during the TRF_300_ time window reflects more faithful encoding which in turn results in better recall during the CT trials.

In our view, the debate whether strong envelope tracking in older adults should be considered beneficial or hindering stems at least partly from the heterogeneity of methods employed to measure envelope tracking. From auditory steady‐state responses over envelope reconstruction (backward modeling) to TRFs (forward modeling), these methods all highlight different aspects and stages of envelope tracking. While forward modeling allows the investigation of envelope tracking as it unfolds over time, envelope reconstruction/backward modeling has the benefit of providing an actual quantification of reconstruction accuracy. We wish to emphasize that for the case of forward modeling, stronger target speaker envelope tracking seems to be beneficial for speech intelligibility, both in younger (Ding & Simon, 2013) and older adults (the present study). Given that with the FUEL, current theory on effortful listening views selective attention as an essential ability for successful speech‐in‐noise processing (Pichora‐Fuller et al., 2016), our finding that envelope tracking is enhanced in participants with better selective attention provides another cue for a beneficial role of envelope tracking. Furthermore, we showed that stronger envelope tracking in multi‐talker babble noise was advantageous even further downstream during speech processing, namely, at the level of memorizing the content of the speech signal. Therefore, the benefits of selective attention and enhanced envelope tracking may well extend beyond object formation and object selection (Shinn‐Cunningham, 2008; Shinn‐Cunningham & Best, 2008) and remove cognitive load during later stages of speech processing (McCoy et al., 2005).

## 5 CONCLUSION

This study investigated the influences of EM and IM on speech‐in‐noise processing in older adults. There was no additive effect of EM and IM on speech processing, but EM influenced how well participants could follow a target speaker in the presence of background talkers. Further, both EM and IM influenced how well participants could memorize the content of the target speaker's speech. The amount of speech envelope tracking was affected by IM and modulated by selective attention. Also, the amplitude of a later component of the TRF to the speech envelope, the TRF_300_, was positively related to how well participants could memorize the content of the target speaker's speech. To summarize, increases in EM and IM both rendered speech‐in‐noise processing more difficult, and affected speech envelope processing.

## CONFLICT OF INTEREST

The authors have no conflict of interest to declare.

## Data Availability

The data that support the findings of this study are available on request from the corresponding author. The data are not publicly available due to privacy or ethical restrictions.
